# Adolescent sexual behaviour in a refugee setting in Uganda

**DOI:** 10.1186/s12978-021-01181-0

**Published:** 2021-06-24

**Authors:** Paul Bukuluki, Peter Kisaakye, Hadijah Mwenyango, George Palattiyil

**Affiliations:** 1 Department of Social Work and Social Administration, School of Social Sciences, Makerere University, Kampala, Uganda; 2 Department of Population Studies, School of Statistics and Planning, Makerere University, Kampala, Uganda; 3 Social Work, School of Social and Political Science, The University of Edinburgh, Scotland, UK

**Keywords:** Adolescents, Refugees, Sexual behaviour, Uganda

## Abstract

**Background:**

Children under 18 years old constituted more than half (52%) of the refugee population in 2017. Adolescent Sexual and reproductive health is an essential component of primary health care. Yet, not every refugee adolescent is able to access sexual and reproductive health services.

**Methods:**

Using quantitative data from 356 refugee adolescents and qualitative data (17 in-depth interviews and nine key informant interviews), we examine refugee adolescent sexual behaviour in Bidibidi settlement—the largest refugee settlement in Uganda using a binary logistic regression model.

**Results:**

The results show that 25% of refugee adolescents in Bidibidi refugee settlement had ever had sex. After controlling for all factors, results show that refugee adolescents aged 16–18 years (OR  =  3.47; 95% CI  =  1.09–10.94), males (OR  =  17.59; 95% CI  =  4.48–69.07), not in school (OR  =  14.57; 95% CI  =  2.20–96.35) were more likely to engage in sexual behaviour than their counterparts. Refugee adolescents who do not agree that a girl cannot get pregnant if she has sex while standing up (knowledge about getting pregnant) were significantly less associated with sexual behaviour (OR  =  0.30; 95% CI  =  0.10–0.85).

**Conclusions:**

Results from this study show that keeping refugee adolescents in school and providing sexual and reproductive health information are likely to delay refugee adolescents’ engagement in sexual behaviour. Therefore, there is need to promote keeping refugee adolescents in school in order to improve sexual and reproductive health of adolescent refugees living in low-income countries such as Uganda.

## Plain language summary

Sexual and reproductive health is an essential component of primary health care. Limited access to sexual and reproductive health (SRH) services for adolescent refugees particularly in low income countries can affect their reproductive health. Similarly, limited knowledge of the determinants of sexual behaviour of adolescent refugees in low income countries puts them at risk of SRH challenges.

This study uses quantitative data from 356 adolescent refugees and qualitative (17 in-depth interviews with adolescent refugees and nine key informant interviews with service providers) to examine the factors that influence the sexual behaviour of adolescent refugees in Bidibidi refugee settlement.

The findings show that older age, male sex, not being in school and having knowledge of getting pregnant were associated with sexual behaviour among adolescent refugees in Bidibidi refugee settlement in Uganda.

There is need to promote keeping refugee adolescents in school and providing them with relevant SRH information to contribute to improving sexual and reproductive health of adolescent refugees living in low-income countries such as Uganda.

## Introduction

During 2019, an estimated 11.0 million people were newly displaced [1]. Globally, adolescents form a substantial proportion of the most vulnerable population in refugee situations [2, 3]. In 2017, children under 18 years old constituted more than half (52%) of the refugee population [3]. We define adolescence as the period between 10 and 19 years of age [4] or a period between childhood and adulthood [5]. This period involves a process of growing to maturity and is characterised with physical, cognitive, behavioural and psychosocial changes [6]. Sometimes adolescents become autonomous which leads to increased decision making some of which could be risky [7, 8]. For instance, some adolescents start having sex and experimenting with substances such as alcohol, tobacco or drugs [9]. Others start having sex as a result of exposure to pornographic material [10, 11] or due to coercion [12]. Adolescents are categorised as a vulnerable group particularly to sexual violence that result into unintended pregnancy, school dropouts, unsafe abortions and STIs, including HIV [4, 13–15]. According to the Inter-Agency Working Group for Reproductive Health in Crises (IAWG), 1.2 million adolescents die annually because of complications from pregnancy/ birth—around the world and two-thirds of these deaths occur in the least developed countries in Africa and Southeast Asia [3]. This is due to inadequate or lack of sexual and reproductive health information and services. Under international law, adolescents have rights through the Convention on the Rights of the Child (CRC) until they reach 18 years of age. Examples of rights protected in the CRC include rights to health, life, education and information, protection from sexual exploitation, the right to be free from discrimination, violence and harmful practices.

Sexual and reproductive health is an essential component of primary health care. Sexual and Reproductive Health (SRH) rights include the right to have access to sexual and reproductive health care and information and autonomy in sexual and reproductive decision-making [6]. This involves voluntary, informed, and affordable family planning services; pre-natal care, safe motherhood services, assisted childbirth from a trained attendant, prevention and treatment of sexually transmitted infections (STIs), including HIV and AIDS and cervical cancer, prevention and treatment of violence against women and girls, safe and accessible post-abortion care and sexual health information, education, and counselling [6]. However, not every adolescent is able to access these services. For instance, adolescents in low income settings face restricted access to SRH information and services, inadequate nutrition, and limited access to healthcare which impacts their welfare [3]. Even when adolescent SRH services are available, it is not automatic that adolescents benefit from these [5]. For instance, it is not obvious that service providers are aware of adolescents’ specific needs.

In humanitarian settings, adolescent sexual and reproductive health (ASRH) needs are either ignored or neglected [3]. Uganda hosts the third-largest refugee population in the world, and the largest in Africa [16–18]. By May 2020 Uganda was host to an estimated 1.4 million refugees and asylum seekers [19]. These are largely hosted in the West Nile (in Moyo, Adjumani, Yumbe and Arua districts), Northern (Lamwo district) and Western (Isingiro, Kikube, Kiryandongo districts) regions of the country [16]. The majority of refugees in Uganda are from South Sudan and the Democratic Republic of Congo [20]. The majority of the refugees (82 percent) are women and children [21]. Humanitarian settings in Uganda are associated with high prevalence of gender based violence (GBV) which is a risk factor for poor ASRH outcomes [22]. In Uganda, ASRH services such as HIV and AIDS, and contraceptive use are often neglected. Moreover, access and availability to ASRH is limited due to social and structural factors and over-burdened health systems within humanitarian settlements [23]. For example in Nakivale refugee settlement, refugee adolescents are reported to engage in early sexual intercourse in exchange for money [18, 24, 25].

Refugee adolescent refugees face added challenges due to displacement, stigma and discrimination [26]. The SRH needs of adolescent refugees are exacerbated by disruption of family and social structures, gender imbalances between men and women, violence and poverty and its effects such as psychological disorders and mental health needs [3]. These result in higher risks of sexual abuse, exploitation and violence, and cause transmission of sexually transmitted infections and/or unwanted pregnancies, unsafe abortions and early marriage [3, 27–29]. Research also indicates that adolescent refugee girls are at increased risk of death from pregnancy and childbirth complications [3]. On the other hand, adolescent boys especially the unaccompanied, face vulnerabilities such as sexual abuse, exploitation, violence, aggressive and risky behaviours (such as alcohol and drug use) [3]. Despite the marked difference in SRH by gender, most studies tend to focus more on girls than boys [30]. This study examined adolescents’ sexual behaviour in Bidibidi settlement in Uganda. It is important to understand the SRH situation of refugee adolescents to develop a plan that responds to their specific needs especially in countries with limited resources to tackle all needs at once.

### Adolescent sexual and reproductive health (ASRH)

The United Nations Commissioner for Refugees (UNHCR) considers ASRH as a state of complete physical, mental and social well-being, not merely the absence of disease and infirmity, in all matters relating to the reproductive system and to its functions and processes specifically applied to adolescents [4]. On the other hand, sexuality comprises of sex, gender identities and roles, sexual orientation, eroticism, pleasure, intimacy and reproduction [4]. These are expressed or experienced differently depending on biological, psychological, social, economic, political, cultural, ethical, legal, historical, religious and spiritual factors [4]. Adolescents need comprehensive SRH services to productively transit from childhood to adulthood. In most situations this is not realised due to a lack of proper guidance from adult role models, existing social norms and structures and community influences which might be restrictive [31]. Generally, adolescents face challenges in accessing RH services for instance individual barriers (such as shame, anxiety about RH, lack of awareness about available services and poor health seeking behaviours); socio-cultural barriers (such as prescriptive social norms, stigma surrounding sexually active adolescents, cultural barriers, illiteracy and poor attitude of service providers); and structural barriers (such as long distances to health facilities, lack of health facilities and medical products, inconvenient hours of operation, long queues and lack of confidentiality). These are worsened for those in refugee situations. Sometimes, it is common to overly focus on the needs of girls and neglect boys. However, adolescent boys need to be empowered and catered to improve the situation of girls. Moreover, adolescent boys are more likely than adolescent girls to have had sex [32]. For instance, it is stated that boys face high rates of STI, HIV and AIDS, and therefore provision of sexual health information to them lowers high-risk sexual behaviour [5]. UNFPA and Save the children recommend the Minimum Initial Service Package (MISP) as an effective tool for providing acceptable and inclusive RH services in humanitarian situations [5]. According to MISP, SRH interventions must prioritise prevention and management of the consequences of sexual violence, avert excess new-born and maternal morbidity and mortality, reduce HIV transmission, must be coordinated and comprehensive [33]. Adolescents in humanitarian settings are at a higher risk of experiencing gender-based violence (GBV), STIs, mental stress, sexual coercion, rape, forced prostitution, human trafficking than adolescents in development settings [5]. Such conditions make refugee adolescents more vulnerable to engage in sexual behaviour in an environment where they may not have access to ASRH services. While these conditions are common in humanitarian settings, the risk factor profile of refugee adolescents and how these are likely to affect their sexual behaviour is not adequately researched. Similarly, refugee adolescents are not a homogenous group and refugee settlements are dynamic—warranting continuous research and assessment. Therefore, this paper seeks to answer the following research question: how do socio-economic, demographic and SRH knowledge factors influence adolescent sexual behaviour in a humanitarian setting? While several studies on ASRH behaviour have been undertaken in development settings, few studies have explored ASRH behaviour in humanitarian settings in low-income countries such as Uganda [34–36]. We draw on Bidibidi refugee settlement—the second largest refugee settlement in the world—to fill this gap by examining the socio-economic, demographic and SRH knowledge factors that are associated with sexual behaviour. Findings from the study can help to identify the risk factor profiles of adolescents and inform the design of interventions that are sensitive to peculiar characteristics, needs and context of refugee adolescents in humanitarian settings. This study contributes to furthering understanding of the risk factors that are likely to make adolescents living in humanitarian settings susceptible to engaging in sexual behaviour that may affect their ASRH outcomes.

## Material and methods

### Study setting

**Fig. 1:**
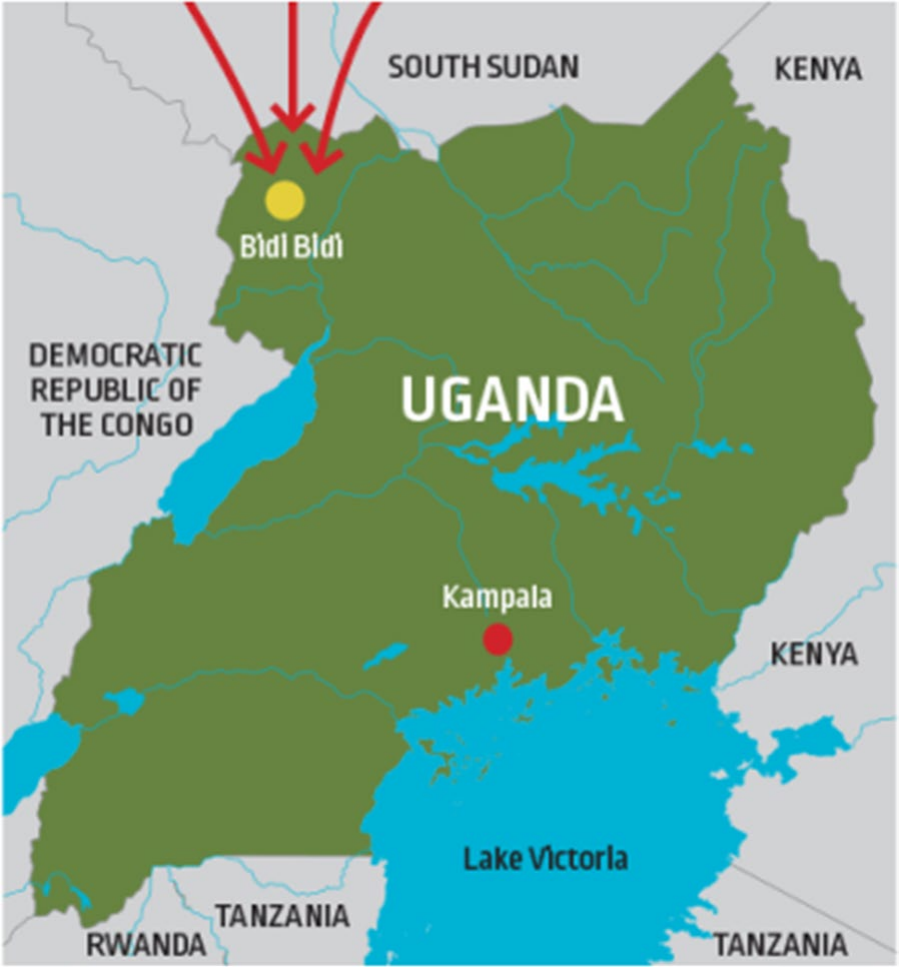
A map of Uganda showing the location of Bidibidi refugee settlement. Source: https://ischp.net/2019/05/01/research-informed-social-enterprises-with-south-sudanese-refugees-in-uganda-a-partnership-project/

Bidibidi Refugee Settlement is located in the West Nile Area of Uganda – part of the greater northern Uganda region (see Fig. 1). The settlement is home to over 270,000 South Sudanese refugees and it is the second largest refugee settlement in the world. This settlement was opened in mid-2016 to cope with a high influx of South Sudanese refugees fleeing from the violent conflict in South Sudan. Refugees in Bidibidi settlement largely depend on subsistence agriculture and support from UNHCR and World Food Programme for their survival and livelihood [37].

### Source of data and sample size

The data used in this paper come from a cross-sectional survey (SRH needs assessment survey 2020) that collected quantitative and qualitative data from Bidibidi refugee settlement in Yumbe district, Uganda. The study site was chosen for inclusion in the study because it is the largest refugee settlement in Uganda. The Quantitative survey collected information from 356 refugee adolescents.

A qualitative approach was used to conduct in-depth and semi-structured interviews. In-depth interviews (IDIs) were used to collect information from adolescent refugees (boys and girls). Key-informant interviews were used to collect information from key stakeholders such as representatives from the Office of the Prime Minister (OPM), Non-Governmental Organisations (NGOs), health facilities, local leaders, volunteers and village health team (VHT) members. In total, the study conducted 17 in-depth interviews (6 boys and 11 girls), and nine key informant interviews (KIIs).

### Data collection and ethical clearance

Data collection was conducted by a team of well-trained research assistants using the Computer Assisted Personal Interviewing (CAPI) technology. With the use of CAPI, information collected was accessed in real time. Training of research assistants was carried out for three days. Prior to the main data collection exercise, a pre-test was also conducted in June 2020 to test the suitability of the instrument, and comments arising from the pre-test were incorporated in the revision of the final questionnaire. The main data collection exercise took place in June–July 2020 immediately after the pre-test.

Participants for the in-depth and semi-structured interviews were interviewed separately, and responses were recorded with an audio recorder with permission from the participant. Given the context of COVID-19, interviews were conducted in cognisant of the Standard Operating Procedures (SOPs), and in some instances, interviewers conducted phone-based interviews considering the ethical guidelines provided by the World Health Organisation (WHO) to adhere to while conducting research during COVID-19, to ensure no harm to the participants. Ethical clearance to conduct the study was granted by the Makerere University School of Social Sciences Institutional Review Board and the Research Ethics Committee at the University of Edinburgh. Respondents were assured of their safety, privacy and confidentiality. All participants who were 18 years and above had to consent to participate in the study. We involved parents and caretakers for permission to involve their adolescents in the study and upon being granted permission, assent was obtained from adolescents below the age of 18 years.

### Study sampling

Cluster sampling technique was used to select three blocks to be included in the study. Three blocks were selected from each block to give a total of nine blocks. A list of all households in each block with adolescents in the age group 13–18 years was developed. Simple random sampling was then used to select respondents for interview from each block.

Purposive sampling—a non-probability sampling technique was used to select participants to answer qualitative questions. This was done in order collect rich information and knowledge about the subject area [38]. Participants were identified with the help of the Refugee Welfare Council 1 (RWC1). During the recruitment of adolescents, participants were purposively selected to include the following categories: young and older adolescents, in school and out of school, married but still in school, married and out of school, unmarried and out of school, unmarried but still in school, pregnant female adolescents and those who have ever given birth, and adolescent single mothers. This information provided a diverse sample to allow depth and breadth in analysis.

### Measurement of variables

#### Dependent variable

We used the question on ever had sex to measure sexual activity of adolescents [39–41]. In this paper, sexual activity means any form of self-reported engagement in sexual intercourse [42]. Adolescents were asked if they have ever had sexual intercourse. The dependent variable is a binary variable because responses to the question were either Yes or No.

### Independent variables

Background characteristics of respondents included age which was collected in single years with the minimum being 13 years and maximum 18 years. We created two age groups 13–15 and 16–18 years. Duration of stay was categorised into two groups: 1–3 years and 4–9 years. All respondents in the study originate from South Sudan. Education attainment is either no education, primary or secondary. Religion was categorised into five groups: Muslim, Catholic, Anglican, Pentecostal, and Other religions. Respondents were either married or unmarried. Current school status was binary: Yes or No.

Questions about SRH knowledge included: can a person get STIs without having sex, knowledge of modern contraception. A response to each of the questions was either ‘Yes’ or ‘No’. Respondents were also asked to mention a type of contraception they think is the most suitable for young people. Adolescents were asked to state whether they have ever visited a health facility for sexual and reproductive health services. Response to this question was either ‘Yes’ or ‘No’.

### Data analysis

Quantitative data analysis was performed using the Stata software version 15 [43]. The distribution of adolescents’ characteristics was presented at the univariate level of analysis. A Pearson-chi-square test was computed to test the association between selected adolescents’ characteristics and ever had sex. A binary logistic regression model was fitted (because the outcome variable is binary) to examine the correlates of ever had sex among adolescents in a refugee setting. Model 1 controlled for only the socio-economic and demographic factors. Model 2 controlled for only sexual and reproductive health knowledge variables and Model 3 controlled for all variables considered in the study. Only variables that were significant at the bivariate level of analysis were included in the model.

The analysis of qualitative data started during fieldwork to identify emerging themes [44]. Qualitative data were first transcribed from the local language, and then translated into English. Thematic analysis was then used to analyse the transcripts. Qualitative data were organised into codes, categories, themes and sub-themes relating to socio-economic, demographic, SRH knowledge factors, and context-specific experiences that are perceived to affect refugee adolescent sexual behaviour in a humanitarian setting [45]. Selected quotes from participants that rhyme with the key themes and sub-themes were extracted and included in the results.

## Results

### Socio-economic and demographic characteristics of refugee adolescents in Bidibidi settlement

The distribution of respondents’ characteristics is shown in Table 1. The results show that the majority were in the age group 13–15 years (60%) and 65% of the respondents were female. About 60% of adolescent refugees had stayed in Uganda for 4–9 years. Most adolescents had primary or no education (81%), unmarried (95%) and were currently in school (90%).

**Table 1:** Distribution of adolescents by socio-economic and demographic characteristics, SRH needs assessment survey 2020

Background characteristics	Number	Percent
*Respondent’s age*		
13–15	214	60.1
16–18	142	39.9
*Sex*		
Female	230	64.6
Male	126	35.4
*Duration of stay in Uganda*		
1–3 years	145	40.7
4–9 years	211	59.3
*Education attainment*		
No education or primary	290	81.5
Secondary	66	18.5
*Religion*		
Other	5	1.4
Muslim	13	3.6
Catholic	178	50.0
Anglican	74	20.8
Pentecostal	86	24.2
*Current marital status*		
Married	17	4.8
Unmarried	339	95.2
*Currently in school*		
Yes	319	89.6
No	37	10.4
Total	356	100

### Sexual and reproductive health knowledge of refugee adolescents in Bidibidi settlement

Table 2 provides information about sexual knowledge of adolescents. Most adolescents agreed that a woman can get sexually transmitted diseases without sexual intercourse (61%). Most adolescents (65%) knew of contraception, for instance one of the adolescents said,

*I know the one that is injected for five years, the one injected for three months—after every three months you go to the facility, I also know about condoms [and]there is one given for 10 years (Ruth, 16-year old)*

**Table 2:** Sexual and reproductive health knowledge, SRH needs assessment survey 2020

Sexual and reproductive health knowledge	Number	Percent
*Can one get an STI without sex*		
Yes	218	61.2
No	138	38.8
*Know of contraception*		
Yes	230	64.6
No	126	35.4
*Contraceptive method most suitable for young people*		
Hormonal (Pill/injectable)	20	8.8
Barrier (condom)	185	81.5
Traditional (abstinence/withdrawal)	22	9.7
*Ever visited a health facility for sexual and reproductive health services*		
Yes	57	16.0
No	299	84.0
Total	356	100

Three refugee adolescents who knew of contraception did not state the contraceptive method that is most suitable for young people

However, the condom was mentioned by adolescents to be the most suitable (81%) method for adolescents during sexual intercourse. Finally, most adolescents (84%) had never visited any health facility for sexual and reproductive services. This was also revealed by some adolescents when asked about availability of community places for young people to learn about their bodies, sex, sexually transmitted infections, contraception, abortion and unplanned pregnancies. They said,

No, it is not there, but I have heard about it that such services are there at the hospital but I have never gone there (Hildah, 13-year-old).

For me, I have never gone there (hospital) but one day my wife went there (Simon 18-year-old)

I have never gone for contraceptives at the health facility (Ruth, 16-year-old)

### Associations between socio-economic and demographic factors and ever had sex among adolescent refugees in Bidibidi settlement

Table 3 shows bivariate relationships of selected background factors of adolescents and ever had sex. The results show that 25% of refugee adolescents in Bidibidi refugee settlement had ever had sex. The results show a significant association between age of the adolescents, sex, education attainment, marital status and current school status. The results show that most respondents (75%) reported not to have ever had sexual intercourse. Table 3 shows that 38% of adolescents in the age group 16–18 years, 51% of males, 23% of adolescents with primary or no education, and 54% of those who were not in school had ever had sex. Results in Table 3 show that refugee adolescents that were married have never had sex. This could be due to separation from the spouse in the process of fleeing from violent conflict. Similarly, marriage in most African settings is a process where consummation of marriage may take a while from the time of commencing the marriage process [46–48]. In this case, couples may regard themselves as married even though they are not engaging in sexual intercourse.

**Table 3:** Background factors and ever had sex among adolescent refugees, SRH needs assessment survey 2020

Background characteristics	Ever had sex		Chi-square (*P*-value)
	Yes	No	
*Age*			20.320 (0.000)
13–15	16.8	83.2	
16–18	38.0	62.0	
*Sex*			67.202 (0.000)
Female	11.3	88.7	
Male	50.8	49.2	
*Duration of stay in Uganda*			0.007 (0.932)
1–3 years	25.5	74.5	
4–9 years	25.1	74.9	
*Education attainment*			3.926 (0.048)
No education/primary	23.1	76.9	
Secondary	34.8	65.1	
*Religion*			5.927 (0.205)
Other	60.0	40.0	
Muslim	23.1	76.9	
Catholic	23.0	77.0	
Anglican	21.6	78.4	
Pentecostal	31.4	68.6	
*Current marital status*			24.765 (0.000)
Married	76.5	23.5	
Unmarried	22.7	77.3	
*Currently in school*			18.097 (0.000)
Yes	21.9	78.1	
No	54.1	45.9	
Total	25.3	74.7	

### Associations between sexual and reproductive health knowledge and ever had sex among adolescent refugees in Bidibidi settlement

Table 4 shows that all the variables considered were significantly associated with ever had sex apart from type of contraceptive method that is suitable for young people. Most adolescents who had ever had sex had visited a health facility for sexual and reproductive health services (44%). Half of adolescents (50%) who had sex were using traditional methods as mentioned by one adolescent,

I only use the natural method my husband has told me. But I have plans of using one of the family planning methods (Ruth, 16-year-old).

**Table 4:** Sexual and reproductive health knowledge and ever had sex, SRH needs assessment survey 2020

Sexual and reproductive health knowledge	Ever had sex		Chi-square (*p*-value)
	Yes	No	
*Can one get an STI without sex*			6.406 (0.011)
Yes	20.6	79.4	
No	32.6	67.4	
*Know of contraception*			20.730 (0.000)
Yes	33.0	67.0	
No	11.1	88.9	
*Contraceptive method most suitable for young people*			4.620 (0.099)
Hormonal (pill/injectable)	20.0	80.0	
Barrier (condom)	31.3	68.7	
Traditional (abstinence/withdrawal)	50.0	50.0	
*Ever visited a health facility for sexual and reproductive health services*			12.401 (0.000)
Yes	43.9	56.1	
No	21.7	78.3	
Total	25.3	74.7	

Table 4 shows that a third (33%) of respondents who had ever had sex knew of a contraceptive method. As mentioned earlier, the most suitable method for young people was condoms. This could be because the condoms were mainly promoted by health providers. This was uncovered in these statements,

*So for our adolescents, we highly encourage them to use condoms since with condoms there is ninety-something kind of assurance that you will be safe if it is used correctly (SRHO, Save the children).*

*The condoms are only given to adult males, the women are not given whether you are an adult or an adolescent, instead they tell the women to use family planning (Hellen, 17-year-old)*

### Determinants of ever had sex among refugee adolescent in Bidibidi settlement

Table 5 shows three models. The first model (Model 1) controlled for only background factors of refugee adolescents. Model 2 controlled for sexual and reproductive health knowledge variables and the last model (Model 3) controlled for all variables in the study.

**Table 5:** Odds ratios from a binary logistic regression model predicting sexual behaviour (ever had sex) among adolescent refugees, SRH needs assessment survey 2020

Variable	Model 1	Model 2	Model 3
*Age (RC = 13–15)*			
16–18	3.16** (1.56–6.41)		2.87** (1.41–5.87)
*Sex (RC = Female)*			
Male	22.92*** (10.77–48.75)		20.23*** (9.20–44.48)
*Education attainment (RC = no education/primary)*			
Secondary	1.44 (0.58–3.56)		1.15 (0.44–2.99)
*Current marital status (RC = unmarried)*			
Married	9.59** (2.19–41.89)		8.72** (1.95–39.07)
*Currently in school (RC = Yes)*			
No	4.90** (1.63–14.75)		4.66** (1.53–14.19)
*Can one get an STI without sex (RC = Yes)*			
No		2. 24** (1.33–3.75)	1.28 (0.68–2.40)
*Know of contraception (RC = Yes)*			
No		0.27*** (0.14–0.52)	0.69 (0.33–1.48)
*Ever visited a health facility for sexual and reproductive health services (RC = No)*			
Yes		2.36** (1.26–4.42)	2.10 (0.89–4.94)
Constant	0.03*** (0.01–0.06)	0.29*** (0.19–0.44)	0.03*** (0.01–0.07)
Number of observations	356	356	356
Likelihood ratio Chi-squared (probability)	134.9 (0.000)	37.9 (0.000)	139.8 (0.000)
Pseudo R-squared value	0.335	0.094	0.347

*RC*  reference category

Confidence intervals in parentheses: *p < 0.05; **p < 0.01; ***p < 0.001

When only background factors are included in the model, results show that adolescents in the age group 16–18 years were more likely (OR  =  3.16; 95% CI  =  1.56–6.41) to have ever had sex than their counterparts in the age group 13–15 years. Males were about 23 times more likely than their female counterparts to have had sex (OR  =  22.92; 95% CI  =  10.77–48.75). This was also observed when asking about questions on condoms. One of the key informants said that the boys collected condoms more than girls:

We do give condoms but they are largely consumed by the male adolescents (Sexual Reproductive Health Officer (SRHO), Save the children).

Another key informant confirmed that girls do not collect condoms and said,

*If they come we give, but I have not seen any single girl coming, it is only boys, every week they come (Clinical Officer Lyete Health Centre III)*

Married adolescents were 9 times more likely than unmarried adolescents to have ever had sex (OR = 9.59; 95% CI = 2.19–41.89). This result may be due to the fact that sexual intercourse mostly happens freely between married couples. However, we are unable to tell from our data whether marriage came before having sex for the first time or after. Adolescents who were currently out of school were more likely (OR = 4.90; 95% CI = 1.63–14.75) to have ever had sex.

When sexual and reproductive health knowledge variables are considered in the model (Model 2), the results in Table 5 show that refugee adolescents who said that one cannot get an STI without having sex were more likely (OR = 2.23; 95% CI = 1.33–3.75 than their counterparts to have ever had sex. Refugee adolescents who do not have knowledge about the use contraception were less likely (OR = 0.27; 95% CI = 0.14–0.52) than their counterparts with knowledge to have ever had sex. Adolescents who had ever visited a health facility for sexual and reproductive health services were more likely (OR = 2.36; 95% CI = 1.26–4.42) to have ever had sex than adolescents who had never visited a health facility.

The results shown in Table 5 indicate that the effect did not change much when all factors were included in the model (Model 3). The likelihood to have ever had sex is still observed to be higher among 16–18 year olds (OR = 2.87; 95% CI = 1.41–5.87), males (OR = 20.23; 95% CI = 9.20–44.48), married (OR = 8.72; 95% CI = 1.95–39.07) as well as those who were not currently in school (OR = 4.66; 95% CI = 1.53–14.19).

## Discussion

This main objective of this paper was to examine adolescents’ sexual behaviour in Bidibidi—the largest refugee setting in Uganda. In this paper, we adopted the widely used measure of sexual behaviour—ever had sex—to measure sexual behaviour of adolescents in Bidibidi refugee setting [39–41]. Our study used mixed methods to provide context to the sexual behaviour of adolescents in refugee settings [30]. This inquiry was motivated by the fact that adolescents form the biggest proportion of the most vulnerable population in refugee settings, particularly in low resource settings [2, 3] yet, comprehensive and quality ASRH information services have not adequately catered for them. Moreover, most studies tend to focus more on females than male adolescents, yet males may have different perspectives and experiences [30]. Our study targeted both male and female adolescents. After controlling for all factors, the results we report in this study show that older adolescents (16–18 years), males, married and adolescents that were not in school were associated with sexual behaviour in Bidibidi settlement.

Older adolescents may have sex because of increased autonomy or peer pressure [7, 8]. Male adolescents were more likely than girls to have had sex. Previous research shows that boys believe or they are told while growing up that they must have sex with girls [32]. This perception may force or make boys to feel that they must have sex at all costs. Adolescents who were not in school were more likely than their counterparts who were in school to have had sex. While this study cannot verify temporality in understanding the relationship between ever had sex and being out school, since we may not know which came first, previous research has shown that having ever had sex with its adverse effects leads to poor grades or even school drop outs during adolescence [13, 14]. On the other hand, adolescents are likely to engage in sexual relations as a result of being out of school [15]. This is more so in the sub-Saharan African (SSA) context where early and child marriage are relatively high, making being out of school among adolescents a risk factor for marriage and therefore early initiation of sex [27, 28].

Understanding the nuances and risk factors of adolescent sexual behaviour in refugee settings in low-income countries is crucial to inform design of effective SRH programmes that respond to humanitarian contexts. Voices and experiences of adolescents therefore need to be considered in research and SRH programming. It is therefore important to design and implement adolescent friendly services that are accessible and acceptable [49–51]. Besides, the community and parents involvement in ASRH programmes is considered as a sustainable approach in supporting adolescents [5]. Some of the adolescent friendly ASRH measures may encompass re-integration of adolescents into families or their communities, creating safe spaces and education activities, awareness raising and sensitization, strong protection measures, social norm change interventions [31] and strengthening community-based approaches to livelihoods [5].

## Conclusion and implications

The results we report in this study show that 25% of refugee adolescents in Bidibidi refugee settlement had ever had sex. However, refugee adolescents aged 16–18 years, males, married or those out of school were more likely to have ever had sex than their counterparts. The findings suggest that being in school is a protective factor against early sexual debut among refugee adolescents. These findings provide context especially for adolescents living in refugee settings. The results in this study call for the need to promote keeping adolescents in school. This study highlights the need to consider risk factors, experiences and the unique context of adolescent refugees in low-income countries in designing adolescent friendly and relevant SRH services.

The findings reported in this study imply that utilisation SRH by refugee adolescents is low in Bidibidi settlement. This could be due to limited availability, awareness and low perception of risk and vulnerability to SRH related poor outcomes such as unwanted or mistimed pregnancy, STIs, HIV and other associated challenges (school drop-outs, early marriage and parenthood).

### Limitations

This study has some limitations worth mention. Information on ever had sex was self-reported. Therefore, the results we report in this study are likely to suffer from desirability bias and over- or under-reporting. For example, some married refugee adolescents could have been shy to report that they have engaged in sexual intercourse because of taboos and social norms related to revealing information about sexual intercourse in some African settings (52, 53). We were unable to ascertain whether marriage came before or after having sex for married adolescents who reported to have ever had sex. Having such information can help to know whether having sex early pushes adolescents into early marriages or whether early marriages inevitably makes adolescents have early sexual relations.

Given the nature of the data we used in this study—that is cross-sectional—we were unable to measure causality. Moreover, we cannot generalise to other refugee settlements since the findings we report in this study are for one refugee settlement (Bidibidi). Finally, the analyses did not benefit from information on sexual pleasure, communication, high risk sexual behaviour such as coerced sex, rape, having sex without using condoms, experience of HIV or STIs—which could limit our understanding of ASRH for refugee adolescents in humanitarian settings.

### Recommendations

Voices and experiences of adolescents should be given priority in designing SRH programmes for humanitarian settings. There is need for service providers and policy makers to be aware of the drivers and risk factors of sexual behaviour among refugee adolescents. Mixed methods studies are helpful in identifying the peculiar experiences and sexual behaviour of adolescents in refugee settings. Future studies should consider involving both female and male adolescents for their programmes to respond to the unique needs and challenges experienced by both gender.

## Data Availability

All data used in this study are available from the corresponding author on reasonable request.

## Abbreviations

ASRH: Adolescent sexual and reproductive health
CAPI: Computer assisted personal interviewing
GBV: Gender-based violence
IAWG: Inter-agency working group for reproductive health in crises
IDIs: In-depth interviews
KIIs: Key informant interviews
MISP: Minimum initial service package
NGOs: Non-Governmental Organisations
OPM: Office of the Prime Minister
RWC1: Refugee Welfare Council 1
SOPs: Standard operating procedures
SRH: Sexual and reproductive health
SSA: Sub-Saharan Africa
STI: Sexually transmitted infection
UBOS: Uganda Bureau of Statistics
UNFPA: United Nations Population Fund
UNHCR: United Nations High Commissioner for Refugees
UNICEF: United Nations Children’s Fund
UNOCHA: United Nations Office for the Coordination of Humanitarian Affairs
VHT: Village Health Team
WHO: World Health Organisation
